# Multiplexed gene expression profiling identifies the FGFR4 pathway as a novel biomarker in intrahepatic cholangiocarcinoma

**DOI:** 10.18632/oncotarget.16951

**Published:** 2017-04-07

**Authors:** Changhoon Yoo, Jihoon Kang, Deokhoon Kim, Kyu-Pyo Kim, Baek-Yeol Ryoo, Seung-Mo Hong, Jung Jin Hwang, Seong-Yun Jeong, Shin Hwang, Ki-Hun Kim, Young-Joo Lee, Klaus P. Hoeflich, Oleg Schmidt-Kittler, Stephen Miller, Eun Kyung Choi

**Affiliations:** ^1^ Department of Oncology, Asan Medical Center, University of Ulsan College of Medicine, Seoul, Korea; ^2^ Asan Institute for Life Science, University of Ulsan College of Medicine, Seoul, Korea; ^3^ Department of Pathology, Asan Medical Center, University of Ulsan College of Medicine, Seoul, Korea; ^4^ Institute for Innovative Cancer Research, Asan Medical Center, University of Ulsan College of Medicine, Seoul, Korea; ^5^ Department of Surgery, Asan Medical Center, University of Ulsan College of Medicine, Seoul, Korea; ^6^ Blueprint Medicines Corporation, Cambridge, Massachusetts, USA; ^7^ Department of Radiation Oncology, Asan Medical Center, University of Ulsan College of Medicine, Seoul, Korea

**Keywords:** cholangiocarcinoma, intrahepatic, FGF19, FGFR4

## Abstract

**Background:**

The fibroblast growth factor receptor 4 (FGFR4) pathway is an essential regulatory component of bile acid synthesis, and its relationship with hepatocellular carcinoma (HCC) has been reported. We investigated the gene expression and clinical significance of FGFR4 and related pathways in intrahepatic cholangiocarcinoma (iCCA).

**Results:**

The median age was 56 years (range 30–78) and 34 patients (74%) were male. Six patients (13%) had hepatitis B virus infection, with or without liver cirrhosis. Overall survival was significantly associated with *FGFR4* (*p* = 0.004), *FGF19* (*p* = 0.047), *FGF21* (*p* = 0.04), and *KLB* (*p* = 0.03) expression. In the multivariate analysis with potential prognostic factors, high expression of *FGF19*, *FGF21*, and *FGFR4* was significantly associated with better survival. In the analysis using the TCGA iCCA dataset, mRNA overexpression of at least 1 of the FGFR4-related genes was significantly associated with better disease-free survival (*p* = 0.02).

**Materials and Methods:**

We assessed the expression of 98 genes in formalin-fixed paraffin embedded tumor tissue specimens from 46 patients with surgically resected iCCA using a NanoString platform. This included 10 FGF pathway genes (e.g. *FGFR1-4, KLB, FGF3, 4, 19, 21*, and *23*), 19 distal marker genes (e.g. *CYP7A1* and *CYP17A1*), 31 genes relevant to HCC and iCCA (e.g. *AFP*, *TS*), 18 copy number variation matched genes, and 20 control genes. Log-transformation of gene expression was performed for normalization and statistical analysis. Overall survival was correlated with gene expression (< median vs. ≥ median) using a log-rank test. The prognostic impact of FGFR4-related genes was validated using the public TCGA dataset for iCCA.

**Conclusions:**

Our results indicate that mRNA expression of FGFR4-related genes may be a biomarker to define the distinctive molecular phenotype of iCCA. Future preclinical and clinical validation is required to define the role of the FGFR4 pathway in iCCA.

## INTRODUCTION

Biliary tract cancer (BTC) is a heterogeneous group of diseases that includes intrahepatic and extrahepatic cholangiocarcinoma (CCA) and gallbladder cancer. BTC is a rare malignancy and approximately 10,000 new cases are diagnosed annually in the United States and Europe [1]. Surgical resection is the only curative treatment modality for localized disease; however, most patients have a very poor prognosis, as the 5-year overall survival (OS) rate of advanced BTC is approximately 10% [2]. Although the combination of gemcitabine and cisplatin has been established as the standard first-line chemotherapy based on the success of the ABC-02 trial, its success is limited, with median survival of less than 1 year (11.7 months). This highlights the urgent need to develop novel treatments for BTC [3]. Although several agents targeting the EGFR and VEGFR pathways were evaluated in randomized trials, no agent has resulted in a survival benefit in patients with advanced BTC. However, most previous studies were conducted in an unselected patient population with a limited understanding of the biological characteristics of BTC. To enhance survival outcomes, novel agents targeting molecularly defined subgroups of patients may be the key for success, considering the very heterogeneous clinical features of BTC.

Recently, our understanding of the genetic landscape of BTC has improved with the wide application of next generation sequencing (NGS) [4, 5]. Studies indicate that significant proportions of BTC have potentially targetable genetic alterations; however, they also show that there is no single predominant target due to the large heterogeneity within the genetic landscape of BTC. These studies have suggested that potential therapeutic targets differ according to the tumor locations [5]. In intrahepatic CCA (iCCA), fibroblast growth factor receptor 2 *(FGFR2)* fusion, *IDH1/2, BAP1*, and *ARID1A* alterations have been considered as candidates for targeted therapy, and several trials are evaluating the therapeutic implications [6].

The FGFR pathway is involved in cell development, differentiation, survival, migration, and angiogenesis, and may also affect tumorigenesis [7]. In humans, there are 4 FGFRs, which are typical tyrosine kinase receptors (FGFR1-4) and 18 fibroblast growth factors (FGFs), which are ligands for FGFRs. FGF19 is involved in bile acid synthesis and gall bladder filling, and binds to FGFR4. Klotho-beta (KLB) is a transmembrane protein that acts as a cofactor for increased activation of FGFR4 [8]. There is growing evidence that the FGFR4 pathway may contribute to the development of hepatocellular carcinoma (HCC) [9, 10], and selective FGFR4 inhibitors have shown remarkable anti-tumor activity in HCC xenografts harboring *FGF19*-overexpression. Based on these results, a clinical trial of selective FGFR4 inhibitors is ongoing for patients with *FGF19*-amplified or overexpressed HCC [11]. Because iCCA shares many risk factors with HCC, we performed gene expression profiling analysis of iCCA to define the potential relevance of FGFR4 pathway as a therapeutic target.

## RESULTS

### Clinical outcomes

Clinicopathological characteristics of 46 patients with iCCA are presented in Table 1. Underlying liver cirrhosis was present in 3 patients (7%). Recurrence was observed in 30 patients (65%), and 32 patients (70%) had died by the time of analysis. The median follow-up duration was 25.2 months (range, 3–120 months), the median relapse-free survival time was 11.1 months (95% confidence interval [CI], 7.7–14.5 months), and the median OS was 27.9 months (95% CI, 13.1–42.7 months).

**Table 1:** Clinicopathological characteristics

Characteristics	*N* (%), total *n* = 46
**Age**, median (range), years	50 (30–78)
**Sex**	
Male	34 (74%)
Female	12 (26%)
**T stage**	0.69
T1–2	25 (55%)
T3–4	21 (45%)
**N stage**	
N0 or Nx	21 (67%)
N1	15 (33%)
**Resection margin status**	
**R0 resection**	37 (80%)
**R1 resection**	9 (20%)
**Differentiation**	
Well or moderately differentiated	38 (82%)
Poorly differentiated	8 (17%)
Underlying viral hepatitis infection or liver cirrhosis	5 (11%)
Adjuvant treatment	11 (24%)

### Expression of FGFR4-related genes

**Figure 1:**
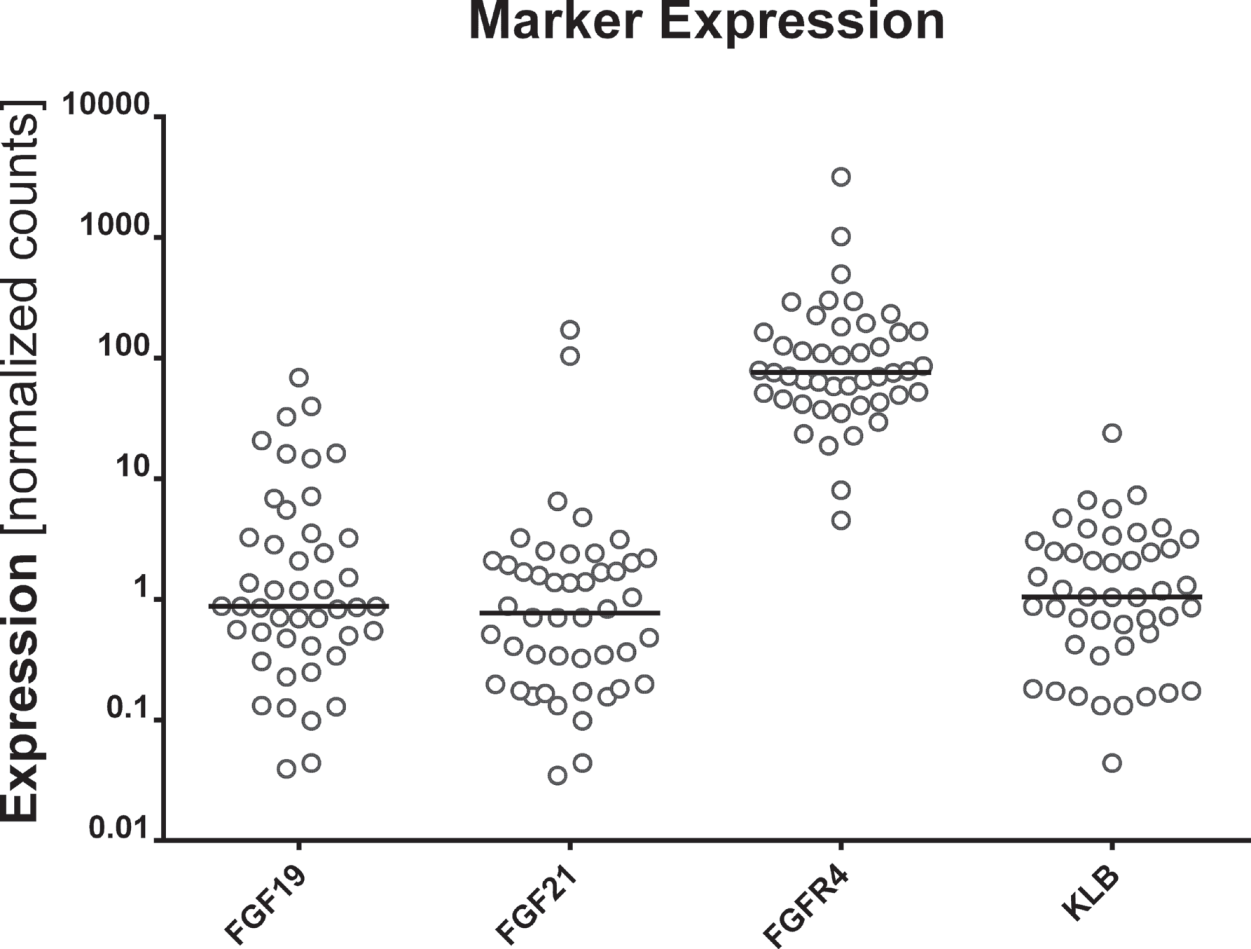
Expression of *FGF19*, *FGF21*, *FGFR*4, and *KLB*

The expression levels of 4 FGFR4-related genes (*FGF19, FGF21, FGFR4*, and *KLB*) were dichotomized according to their median levels for further evaluation (Figure 1). The expression levels of these genes were not associated with clinical characteristics such as age, T stage, N stage, resection margin status, tumor differentiation and adjuvant treatment (*p* > 0.05); however, chronic hepatitis virus infection was associated with high *FGFR4* expression (*p* = 0.049). In the correlative analysis of the expression of each of the 4 genes, there were significant relationships between the expression of *FGF21* and *FGFR4* (*r* = 0.33, *p* = 0.025), and *FGF21* and *KLB* (*r* = 0.47, *p* = 0.001).

### Correlative analysis of survival outcomes

**Figure 2:**
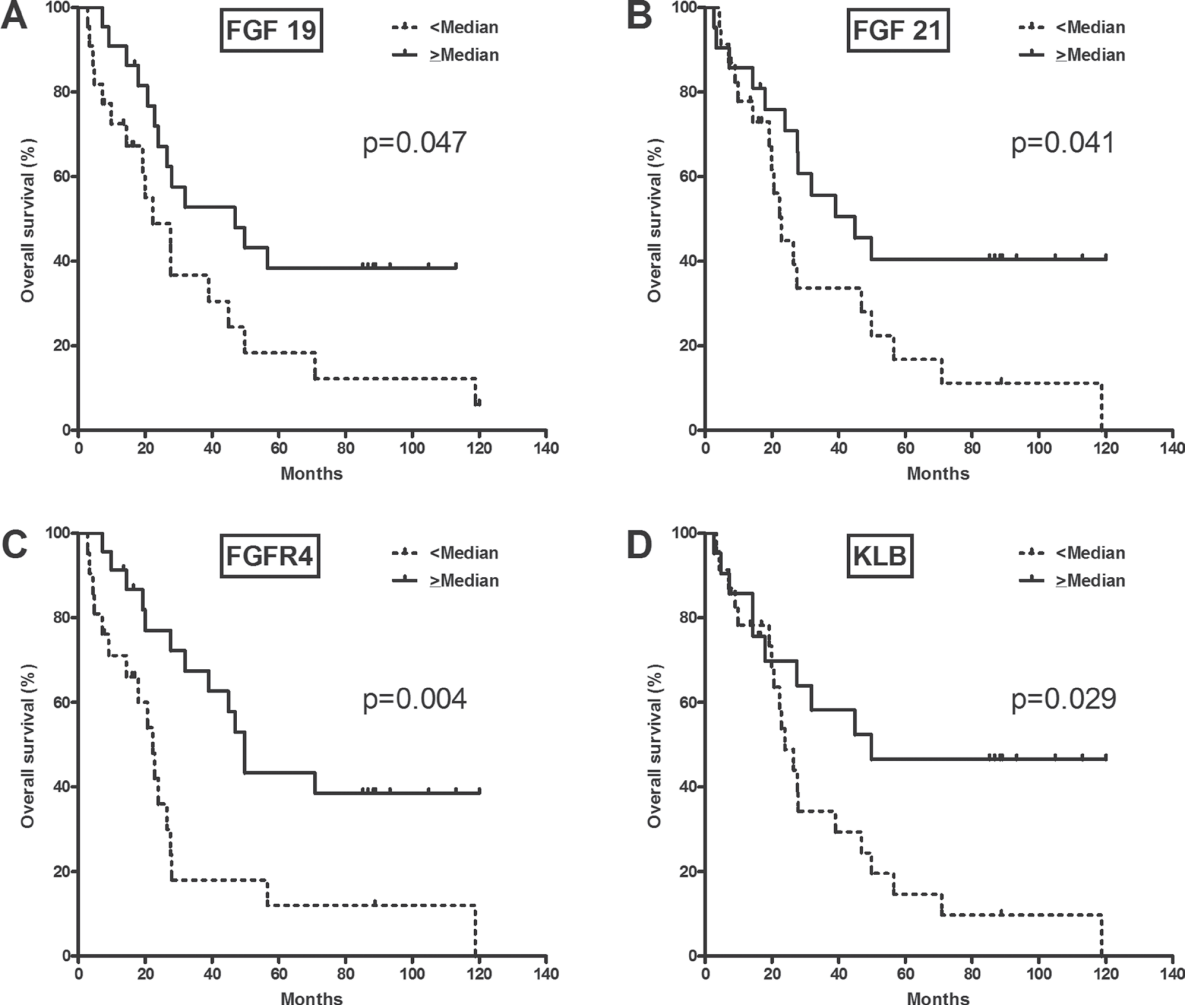
Overall survival according to FGFR4-related gene expression

Correlative analysis between gene expression and OS was performed for 44 patients after exclusion of 2 patients who had died from postoperative complications (Table 2). In univariate analysis, OS was significantly associated with FGFR4-related gene expression, such as *FGF19* (> median vs. < median; unadjusted hazard ratio [HR] 0.48, *p* = 0.047; Figure 2A), *FGF21* (0.47, *p* = 0.041; Figure 2B), *FGFR4* (0.35, *p* = 0.004; Figure 2C), and *KLB* (0.44, *p* = 0.029; Figure 2D). In analyses of the expression of other genes, *CYP8B1* (0.43, *p* = 0.024), *ADH1B* (0.47, *p* = 0.045), *CDKN1B* (0.35, *p* = 0.005), *NCOR1* (0.45, *p* = 0.033), *NF1* (0.36, *p* = 0.006), *SUPV3L1* (0.44, *p* = 0.026), *TIAL1* (0.45, *p* = 0.034), *NROB2* (0.28, *p* = 0.001), *MAP2K3* (0.38, *p* = 0.009), *MYC* (0.47, *p* = 0.040), *CYP2E1* (0.25, *p* < 0.001), and *LGR5* (0.45, *p* = 0.031) were significantly associated with OS.

**Table 2:** Univariate analysis for overall survival

Variables	Hazard ratio (95% CI)	*p*-value
*FGF19*	0.48 (0.23–1.00)	0.047
*FGF21*	0.47 (0.22–0.99)	0.041
*FGFR4*	0.35 (0.17–0.74)	0.004
*KLB*	0.44 (0.20–0.94)	0.029
*CYP8B1*	0.43 (0.20–0.92)	0.024
*ADH1B*	0.47 (0.22–1.00)	0.045
*CDKN1B*	0.35 (0.16–0.74)	0.005
*NCOR1*	0.45 (0.22–0.96)	0.033
*NF1*	0.36 (0.17–0.76)	0.006
*SUPV3L1*	0.44 (0.21–0.92)	0.026
*TIAL1*	0.45 (0.21–0.96)	0.034
*NROB2*	0.28 (0.13–0.60)	0.001
*MAP2K3*	0.38 (0.18–0.81)	0.009
*MYC*	0.47 (0.22–0.98)	0.040
*CYP2EA*	0.25 (0.11–0.56)	< 0.001
*LG5R*	0.45 (0.22–0.95)	0.031

*Abbreviations*: CI = confidence interval.

Multivariate analysis was performed to validate the relevance of FGFR4-related gene expression (*FGF19, FGF21, FGFR4* and *KLB*) as a prognostic factor (Table 3). In the analyses including potential prognostic factors of iCCA, high expression of *FGF19, FGF21*, and *FGFR4* was associated with better OS (*FGF19*, adjusted HR = 0.38 [0.18–0.81], *p* = 0.012; *FGF21*, adjusted HR = 0.41 [0.19–0.89], *p* = 0.024; *FGFR4*, adjusted HR = 0.32 [0.14–0.72], *p* = 0.006). The expression of *KLB* showed a marginal association with OS (adjusted HR = 0.47 [0.20–1.01], *p* = 0.77).

**Table 3:** Multivariate analysis for overall survival according to the expression of FGFR4-related genes

Variables	Hazard ratio (95% CI)	*p*-value
***FGF19***		
***FGF19 (high vs. low)***	***0.38 (0.18–0.81)***	***0.012***
Sex (male *vs*. female)	1.36 (0.52–3.55)	0.53
Age (≥ 60 years *vs*. < 60 years)	0.76 (0.34–1.69)	0.50
N stage (N1 *vs*. N0 or Nx)	1.44 (0.68–3.01)	0.35
Differentiation (poor *vs*. well or moderate)	0.24 (0.07–0.86)	0.029
***FGF21***		
***FGF21 (high vs. low)***	***0.41 (0.19–0.89)***	***0.024***
Sex (male *vs*. female)	1.16 (0.44–3.03)	0.76
Age (≥ 60 years *vs*. < 60 years)	0.73 (0.33–1.60)	0.43
N stage (N1 *vs*. N0 or Nx)	1.84 (0.85–3.99)	0.13
Differentiation (poor *vs*. well or moderate)	0.30 (0.08–1.08)	0.065
***FGFR4***		
***FGFR4 (high vs. low)***	***0.32 (0.14–0.72)***	***0.006***
Sex (male *vs*. female)	1.12 (0.41–3.01)	0.83
Age (≥ 60 years *vs*. < 60 years)	0.95 (0.44–2.16)	0.95
N stage (N1 *vs*. N0 or Nx)	1.90 (0.87–4.16)	0.11
Differentiation (poor *vs*. well or moderate)	0.30 (0.08–1.10)	0.069
***KLB***		
***KLB (high vs. low)***	***0.47 (0.20–1.01)***	***0.077***
Sex (male *vs*. female)	1.11 (0.42–2.94)	0.84
Age (≥ 60 years *vs*. < 60 years)	0.81 (0.37–1.80)	0.61
N stage (N1 *vs*. N0 or Nx)	1.83 (0.83–4.05)	0.14
Differentiation (poor *vs*. well or moderate)	0.40 (0.11–1.46)	0.17

*Abbreviations*: CI = confidence interval.

### Validation using the the cancer genome atlas (TCGA) dataset

**Figure 3:**
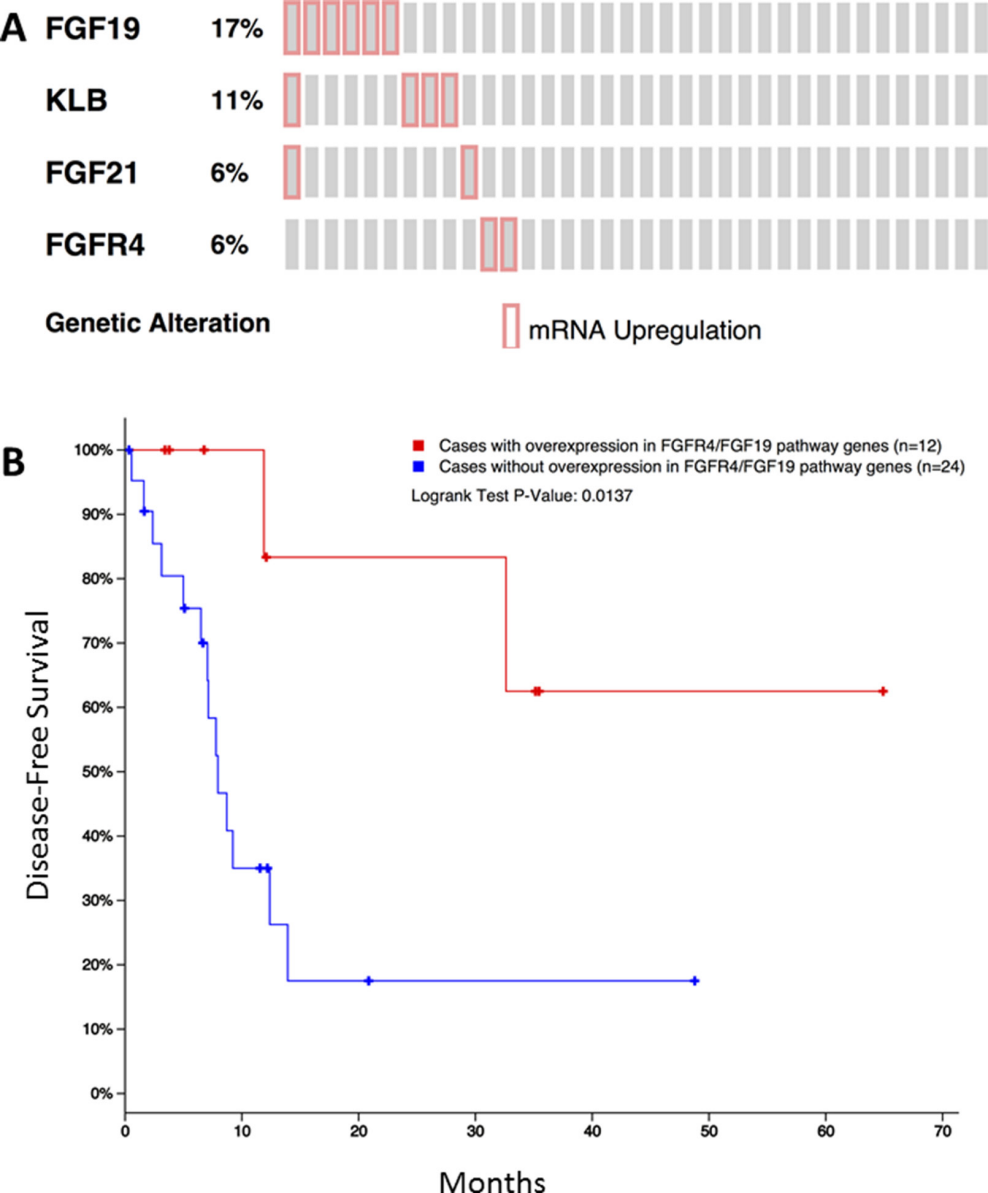
Overexpression of *FGF19*, *KLB*, *FGF21*, and *FGFR4* in the public TCGA dataset for iCCA (**A**) and its impact on disease-free survival (**B**).

The TCGA iCCA dataset includes a total of 36 patients. Overexpression of *FGF19, KLB*, *FGF21*, and *FGFR4* was noted in 6 (17%), 4 (11%), 2 (6%), and 2 (6%) patients, respectively (Figure 3A). Patients who had mRNA overexpression of at least one of *FGF19, FGF21, FGFR4* and *KLB* showed significantly better disease-free survival compared to those without any overexpression in all these genes (*p* = 0.0137, Figure 3B).

## DISCUSSION

Our results show that gene aberrations in the FGFR4 pathway may be a distinct molecular phenotype of CCA, and the prognosis of patients with iCCA may be stratified according to mRNA expression of FGFR4-related genes.

Primary activating *FGFR* aberrations are observed in a variety of cancers, and have been recognized as novel targets for cancer therapy. A previous study based on an NGS assay of 4,853 tumors showed that FGFR aberrations were found in 7.1% of cancers, with the majority being gene amplification (66%), followed by mutation (26%), and rearrangement (8%) [12]. In this study, *FGFR4* was the least affected among the FGFRs, as the frequency of *FGFR4* aberrations was 0.5% across the whole study population. Gene amplification was the most common type of *FGFR4* aberration (78%).

Previous genomic sequencing studies have revealed that *FGFR* gene aberrations are observed in 11–50% of iCCA [5, 13–15]; in contrast, these aberrations are rarely detected in extrahepatic CCA or gallbladder cancer. Although *FGFR1-3* gene rearrangement is well known as the genetic aberration in iCCA, the role of the FGFR4 signaling pathway has not been well elucidated in iCCA. A recent Japanese study showed that *FGF19* gene amplification is detected in 3% of the overall BTC patient population (260 cases, including 145 iCCA), and it was more frequently observed in iCCA compared with extrahepatic CCA and gallbladder cancer [5]. Another Western study which included 377 BTC patients also showed consistent results as the gene aberrations in *FGF19/FGFR4* were observed in 13 patients (3.4%), which consisted of 14% of all detected FGFR pathway gene alterations (*n* = 95) [16].

In our study, when mRNA expression of FGFR4-related genes was dichotomized according to the median expression level, patients with high expression showed better prognosis after surgical resection than those with low expression. This finding was consistent even after adjustment for clinical prognostic factors such as lymph node metastasis, age, and sex. Our results could suggest that the FGFR4 signaling pathway might be the distinct molecular subgroup that could stratify iCCA patients based on their prognosis; however, the reasons for the association between this genetic aberration and a better prognosis is unexplained in this study. Despite this, our results from the analysis using the public TCGA dataset also suggest that mRNA overexpression of FGFR4-related genes may have prognostic implications by indicating better survival outcomes. Our findings are also consistent with the recent study which showed that the BTC patients with FGFR pathway genetic aberrations were significantly associated with improved OS [15]. However, conflicting data exist in terms of the prognostic implication of FGFR4 overexpression in BTC patients. Previous study evaluated the prognostic impact of FGFR4 overexpression in 83 iCCA patients using immunohistochemistry and this study showed that overexpression of FGFR4 was significantly associated with poor prognosis [17]. In addition, *FGF19* overexpression correlated with poor survival outcomes in patients with hepatocellular carcinoma [18]. At this point, however, we could not conclude on the reason for these conflicting results on the prognostic implication of FGFR4: these might be related with different methodology (immunohistochemistry vs mRNA expression using NanoString platform) or cancer types (HCC vs iCCA). Further *in vitro*/*in vivo* studies and biomarker validation analysis in large patient population are needed to define the biological differences in iCCA according to the expression of FGFR4-related genes.

Until recently, the development of targeted agents against iCCA has been mostly focused on *FGFR1-3* gene rearrangement or *IDH1-2* mutations. Our study revealed that FGFR4 may also be a valuable target for the management of patients with iCCA. FGFR inhibitors have been widely investigated in a variety of cancer types using non-selective or selective FGFR-tyrosine kinase inhibitors (TKIs), monoclonal antibodies, and FGF-ligand traps [19]. Therefore, it is necessary to explore the clinical relevance of these FGFR inhibitors in patients with iCCA harboring FGFR4-related gene aberrations to determine whether they effectively inhibit the FGFR4 signaling pathway *in vitro* or *in vivo*. For successful clinical investigation and future development of FGFR4-targeted drugs, however, the FGFR4 pathway-aberrant molecular phenotype should be defined. It is still unclear which markers (DNA, mRNA, or protein) and methodologies are optimal to define the patient population for which FGFR inhibitors are effective [20]. Further studies are needed for this subject.

Several caveats exist in our study. Although we performed extensive mRNA expression analysis of the FGFR pathway, integrative bioinformatics analysis to define the molecular signature grouping could not be performed due to the small number of patients. All patients included in this study had undergone surgical resection. Considering that the clinical behavior of tumors harboring specific genetic aberrations may be different between localized resectable and metastatic disease, our data on prognosis may have some limitations for indicating the impact of high *FGFR4* expression in patients with metastatic iCCA. Considering multiple hypothesis generating compaisons were inevitably done because of the small sample size, there is a possibility that the strength of correlation seen in this study might be overemphasized.

In conclusion, mRNA expression of FGFR4-related genes may define a distinctive molecular phenotype of iCCA. Further validation in the preclinical and clinical settings is needed to determine prevalence and prognostic impact.

## MATERIALS AND METHODS

### Patients

We obtained formalin-fixed paraffin-embedded (FFPE) tumor tissues from 46 patients with histologically confirmed iCCA who underwent curative surgery in Asan Medical Center, Seoul, Korea between July 2005 and March 2011. Tissue samples were obtained from primary tumor (*n* = 45, 98%) and recurrent tumor (*n* = 1, 2%). All procedures were conducted according to the guidelines from the Declaration of Helsinki. Medical records of all included patients were retrospectively reviewed for clinical characteristics and outcomes. The Institutional Review Board at Asan Medical Center approved the protocol.

### NanoString analysis

Total RNA was extracted from 5 to 10 sections of 4-μm thick FFPE sections. Non-tumor elements were removed using manual microdissection guided by H&E-stained slides before transferring to the extraction tube. Total RNA was then extracted using the High Pure RNA paraffin kit (Roche), according to the manufacturer's protocol. A Qubit 3.0 Fluorometer (Thermo Fisher) was used to quantify concentrations of extracted RNA. RNA fragment size and concentration was assessed by an Agilent BioAnalyzer 2100 using the RNA 6000 Pico kit according to manufacturer's protocol. Fragments above 50 bp were integrated to calculate an adjusted RNA concentration. 100 ng of fragment length adjusted RNA was used in NanoString hybridizations.

A NanoString panel was designed to assess 98 genes, including 10 FGF pathway genes (*FGFR1-4*, *KLB*, *FGF3*, *4, 19, 21*, and *23*), 19 response marker genes (*AFP, ABCB11, ACACB, ACOX2, AQP8*, *CYP7A1, CYP7B1, CYP8B1*, *CYP17A1, CYP27A1, EGR1, HMGCR, IGFBP2, LEPR, NR0B2, NRIP1, SCD, SQLE*, and *FRS2*), 31 genes relevant to the oncogenic signaling of HCC and CCA (*AKT1, ARID1A, BAD, BAX, BCL2L1, BIRC5, BRD7, CASP9, CDK1, CDKN1A, CDKN1B, CTNNB1, DAXX, GLUL, HGF, LGR5, MIR21, NCOR1, NF1*, *TGFB1, TSC1, TSC2, ARID2, CDKN3, AURKB, CCNB1, CYP2E1, ADH1B, HGFAC, APOF*, and *FCN2*), 18 copy number variation matched genes (*AXIN1, BICC1, CCND1, CDKN2A, ELL, ERBB2, MAP2K3, MET, MYC, ORAOV1, PTEN, RB1, RECQL4, SAV1, TERT, TP53, TRIM45*, and VEGFA), and 20 control genes (*ACTB, BRAP, CNOT2, COX15, CTCF, EIF2B1, FAM149B1, FAM175B, FBXO18, GAPDH, NRF1, SDHAF2, SF1, SUPT7L, SUPV3L1, TIAL1, VTI1B, WDR33, YY1* and *ZNF143*). The details and brief introduction of the analyzed genes are presented in Supplementary Table 1. Hybridization of probes and total RNA was performed according to the manufacturer's protocol. A NanoString nCounter Digital Analyzer (NanoString Technologies, Seattle, Washington, USA) was used to count the digital barcodes that indicate the number of transcripts. The raw expression data were normalized using nSolver Analysis software. A normalization factor was calculated by obtaining the geometric mean of the positive controls used for each sample and applying it to the raw counts of the nCounter output data to eliminate any variability that was unrelated to the samples. The resulting data were normalized again using the geometric mean of the housekeeping genes. Normalized data were log2 transformed for further analyses.

### Statistical analysis

Gene expression levels were dichotomized according to the median level (< median vs. > median). OS was defined as the time from surgery to death from any cause. We used Kaplan-Meier curves to estimate OS, and compared groups using the log-rank test. Student *t*-test or Fisher's exact test were used to correlate gene expression levels with patient characteristics. Multivariate analyses were conducted using Cox proportional hazards regression model. All results were considered statistically significant if a *p* value of < 0.05 was obtained. SPSS version 21.0 (IBM Co., Armonk, NY) was used for all statistical analyses. Because small sample size may result in overfitting problem in the multivariate model, all potentially related genes and clinical factors in the univariate analyses could not be included in the multivariate analysis. Therefore, we included each FGFR4-related gene (FGF19, FGF21, FGFR4 and KLB) and key clinical prognostic factors (sex, age, lymph node metastasis, and differentiation of tumor) in the multivariate analyses to adjust the impact of potential confounding factors.

### Analysis using the iCCA TCGA dataset

To further evaluate the potential implication of the FGFR4 pathway in iCCA, we analyzed the TCGA gene expression data in the public cBioPortal. Normalized mRNA expression data for iCCA in TCGA was obtained from the portal (http://www.cbioportal.org). The frequency of overexpression (z-score > 2.0) in FGFR4 pathway-related genes, including *FGFR4, FGF19, FGF21*, and *KLB*, was estimated. Survival outcomes were compared according to the overexpression of *FGFR4, FGF19, FGF21*, and *KLB*. All detailed information from the iCCA dataset was available in the public cBioPortal (Cholangiocarcinoma, TCGA, provisional).

## SUPPLEMENTARY MATERIALS TABLE

Supplementary Table 1 Nano String gene list

## Abbreviations

BTC: Biliary tract cancer
iCCA: cholangiocarcinoma
EGFR: epithelial growth factor receptor
VEGFR: vascular endothelial growth factor receptor
FGFR: fibroblast growth factor receptor
HCC: hepatocellular carcinoma
OS: overall survival
CI: confidence interval
KLB: Klotho-beta
TCGA: The Cancer Genome Atlas
TKI: tyrosine kinase inhibitor
