# Supplementary material for: Multiplexed gene expression profiling identifies the FGFR4 pathway as a novel biomarker in intrahepatic cholangiocarcinoma
Source: Oncotarget. 2017 Apr 7;8(24):38592–601. doi: 10.18632/oncotarget.16951 (PMC5503556; doi:10.18632/oncotarget.16951)
Supplement: Supplementary Table 1 [file oncotarget-08-38592-s001.pdf]

Multiplexed gene expression profiling identifies the FGFR4 pathway as a novel biomarker in intrahepatic cholangiocarcinoma

Supplementary Materials

Supplementary Table 1: Nano String gene list. See Supplementary_Table_1
