# Supplementary material for: Multiplexed gene expression profiling identifies the FGFR4 pathway as a novel biomarker in intrahepatic cholangiocarcinoma
Source: Oncotarget. 2017 Apr 7;8(24):38592–601. doi: 10.18632/oncotarget.16951 (PMC5503556; doi:10.18632/oncotarget.16951)
Supplement: Supplementary Table 1 [file oncotarget-08-38592-s002.pdf]

Group	Gene	Entrez_ID	Gene name
**FGFR Pathway integrity**	FGF3	2248	fibroblast growth factor 3
	FGF4	2249	fibroblast growth factor 4
	FGF19	9965	fibroblast growth factor 19
	FGF21	26291	fibroblast growth factor 21
	FGF23	8074	fibroblast growth factor 23
	FGFR1	2260	fibroblast growth factor receptor 1
	FGFR2	2263	fibroblast growth factor receptor 2
	FGFR3	2261	fibroblast growth factor receptor 3
	FGFR4	2264	fibroblast growth factor receptor 4
	KLB	152831	klotho beta
**Response markers**	AFP	174	alpha-fetoprotein
	ABCB11	8647	ATP-binding cassette, sub-family B (MDR/TAP), member 11
	ACACB	32	acetyl-CoA carboxylase beta
	ACOX2	8309	acyl-CoA oxidase 2, branched chain
	AQP8	343	aquaporin 8
	CYP17A1	1586	cytochrome P450, family 17, subfamily A, polypeptide 1
	CYP27A1	1593	cytochrome P450, family 27, subfamily A, polypeptide 1
	CYP7A1	1581	cytochrome P450, family 7, subfamily A, polypeptide 1
	CYP7B1	9420	cytochrome P450, family 7, subfamily B, polypeptide 1
	CYP8B1	1582	cytochrome P450, family 8, subfamily B, polypeptide 1
	EGR1	1958	early growth response 1
	HMGCR	3156	3-hydroxy-3-methylglutaryl-CoA reductase
	IGFBP2	3485	insulin-like growth factor binding protein 2
	LEPR	3953	leptin receptor
	NR0B2	8431	nuclear receptor subfamily 0, group B, member 2
	NRIP1	8204	nuclear receptor interacting protein 1
	SCD	6319	stearoyl-CoA desaturase
	SQLE	6713	squalene epoxidase
	FRS2	10818	fibroblast growth factor receptor substrate 2
**Oncogenic signaling**	AKT1	207	v-akt murine thymoma viral oncogene homolog 1
	ARID1A	8289	AT rich interactive domain 1A (SWI-like)
	BAD	572	BCL2-associated agonist of cell death

	BAX	581	BCL2-associated X protein
	BCL2L1	598	BCL2-like 1
	BIRC5	332	baculoviral IAP repeat containing 5
	BRD7	29117	bromodomain containing 7
	CASP9	842	caspase 9, apoptosis-related cysteine peptidase
	CDK1	983	cyclin-dependent kinase 1
	CDKN1A	1026	cyclin-dependent kinase inhibitor 1A
	CDKN1B	1027	cyclin-dependent kinase inhibitor 1B
	CTNNB1	1499	catenin (cadherin-associated protein), beta 1, 88kDa
	DAXX	1616	death-domain associated protein
	GLUL	2752	glutamate-ammonia ligase
	HGF	3082	hepatocyte growth factor (hepapoietin A; scatter factor)
	LGR5	8549	leucine-rich repeat containing G protein-coupled receptor 5
	MIR21	406991	microRNA 21
	NCOR1	9611	nuclear receptor corepressor 1
	NF1	4763	neurofibromin 1
	TGFB1	7040	transforming growth factor, beta 1
	TSC1	7248	tuberous sclerosis 1
	TSC2	7249	tuberous sclerosis 2
	ARID2	196528	AT rich interactive domain 2 (ARID, RFX-like)
	CDKN3	1033	cyclin-dependent kinase inhibitor 3
	AURKB	9212	aurora kinase B
	CCNB1	891	cyclin B1
	CYP2E1	1571	cytochrome P450, family 2, subfamily E, polypeptide 1
	ADH1B	125	alcohol dehydrogenase 1B
	HGFAC	3083	HGF activator
	APOF	319	apolipoprotein F
	FCN2	2220	ficolin (collagen/fibrinogen domain containing lectin) 2
**CNV matched**	AXIN1	8312	axin 1
	BICC1	80114	BicC family RNA binding protein 1
	CCND1	595	cyclin D1
	CDKN2A	1029	cyclin-dependent kinase inhibitor 2A
	ELL	8178	elongation factor RNA polymerase II

	ERBB2	2064	erb-b2 receptor tyrosine kinase 2
	MAP2K3	5606	mitogen-activated protein kinase kinase 3
	MET	4233	MET proto-oncogene, receptor tyrosine kinase
	MYC	4609	v-myc avian myelocytomatosis viral oncogene homolog
	ORAOV1	220064	oral cancer overexpressed 1
	PTEN	5728	phosphatase and tensin homolog
	RB1	5925	retinoblastoma 1
	RECQL4	9401	RecQ protein-like 4
	SAV1	60485	salvador family WW domain containing protein 1
	TERT	7015	telomerase reverse transcriptase
	TP53	7157	tumor protein p53
	TRIM45	80263	tripartite motif containing 45
	VEGFA	7422	vascular endothelial growth factor A
**Controls**	ACTB	60	actin, beta
	BRAP	8315	BRCA1 associated protein
	CNOT2	4848	CCR4-NOT transcription complex, subunit 2
	COX15	1355	COX15 homolog, cytochrome c oxidase assembly protein (yeast)
	CTCF	10664	CCCTC-binding factor (zinc finger protein)
	EIF2B1	1967	eukaryotic translation initiation factor 2B, subunit 1 alpha, 26kDa
	FAM149B1	317662	family with sequence similarity 149, member B1
	FAM175B	23172	family with sequence similarity 175, member B
	FBXO18	84893	F-box protein, helicase, 18
	GAPDH	2597	glyceraldehyde-3-phosphate dehydrogenase
	NRF1	4899	nuclear respiratory factor 1
	SDHAF2	54949	chromosome 11 open reading frame 79
	SF1	7536	splicing factor 1
	SUPT7L	9913	suppressor of Ty 7 (S. cerevisiae)-like
	SUPV3L1	6832	suppressor of var1, 3-like 1 (S. cerevisiae)
	TIAL1	7073	TIA1 cytotoxic granule-associated RNA binding protein-like 1
	VTI1B	10490	vesicle transport through interaction with t-SNAREs homolog 1B (yeast)
	WDR33	55339	WD repeat domain 33
	YY1	7528	YY1 transcription factor
	ZNF143	7702	zinc finger protein 143
